# Hepatitis D Virus Pathogenesis: A Sense of Complications

**DOI:** 10.3390/v18030278

**Published:** 2026-02-24

**Authors:** Yann Haennel, Thomas F. Baumert, Joachim Lupberger

**Affiliations:** 1 Institute of Translational Medicine and Liver Diseases (ITM), University of Strasbourg, Inserm, UMR_S1110, 67000 Strasbourg, France; yann.haennel@etu.unistra.fr (Y.H.); thomas.baumert@unistra.fr (T.F.B.); 2 Gastroenterology and Hepatology Service, Strasbourg University Hospitals, 67000 Strasbourg, France; 3 Institut Hospitalo-Universitaire (IHU), 67000 Strasbourg, France; 4 Institut Universitaire de France (IUF), 75005 Paris, France

**Keywords:** HDV, pathogenesis, viral/host interactions, immune responses, cancer risk, HCC

## Abstract

Hepatitis D virus (HDV) is a satellite RNA virus of the hepatitis B virus (HBV) infecting an estimated 12 million people worldwide. Chronic HDV infection is causing the most severe form of chronic viral hepatitis, leading to a rapid progression of chronic inflammation to fibrosis, cirrhosis, liver decompensation and cancer. The detailed mechanisms responsible for HDV pathogenicity and its contribution to the development of hepatocellular carcinoma (HCC) are not clearly understood. This review aims to summarize the current knowledge of HDV-induced injuries, which gradually accumulate and increase the oncogenic pressure in the liver. Here, we provide a comprehensive yet concise overview of the following topics: (1) virus sensing and innate responses, (2) molecular basis of HDV pathogenesis, and (3) pathogenesis of chronic HDV infection in patients. We summarize the compelling evidence of the direct and indirect contributions of HDV to the development of HCC, which is driven by the rapid progression to liver cirrhosis. These results led to the classification of HDV as a group 1 carcinogenic agent in 2025 and emphasize the urgent need for improved antiviral and chemopreventive treatments. In addition, it highlights the necessity of routine HDV screening in patients with chronic hepatitis B and intensified HCC surveillance in patients with chronic hepatitis D.

## 1. Hepatitis D Virus

The first mention of the hepatitis D virus (HDV) in scientific literature occurred with the discovery of the delta antigen (HDAg) nearly 50 years ago in patients with chronic hepatitis B (CHB). This viral protein was initially thought to represent a previously unrecognized hepatitis B virus (HBV) antigen [1]. Shortly after, studies revealed that HDAg can be found in two isoforms, as small (S-HDAg) and large (L-HDAg) versions of the same protein, which differ by 19–20 amino acids in size due to RNA editing of the stop codon of S-HDAg during replication [2]. S- and L-HDAg are associated with a circular single-stranded RNA that constitutes the ribonucleoprotein, but it does not code for a capsid or surface protein. Therefore, it can be seen as a defective virus that satellites HBV infection, hijacks the HBV envelope proteins (HBsAg), and thus maintains its own viral cycle. Consequently, HDV shares the viral envelope and the host entry mechanism with HBV (reviewed in detail by [3]). However, HDV particles are not secreted via the multivesicular body pathway like HBV virions [4], but rather through the constitutive secretory pathway via the ER-Golgi intermediate compartment (ERGIC). This difference reflects the composition of its envelope (L-HBs, M-HBs, S-HBs), as HDV-associated HBsAg exhibit a similar stoichiometry as spherical subviral particles of HBV, resulting in more efficient viral packaging than that of complete HBV particles [5]. HDV particles enter hepatocytes through binding of the myristoylated N-terminal region of the pre-S1 domain of the large hepatitis B surface antigen (L-HBsAg) to the sodium/taurocholate co-transporting polypeptide (NTCP), a bile acid transporter expressed at the basolateral membrane of hepatocytes [6,7]. Importantly, HDV can also spread via NTCP-independent mechanisms such as by exosomal transfer [8]. As HBsAg has similar density characteristics to high-density lipoprotein (HDL) [9] it is conceivable that the HDL salvage pathway may promote HDV propagation. Genetic diversity among HDV isolates has led to the classification of eight distinct genotypes (HDV-1 to HDV-8) [10], which differ substantially in their replication capacities [11]. Globally, HDV infection affects an estimated 4.5% (95% CI 3.6–5.7) of HBsAg-positive patients, corresponding to ~12 million cases [12]. Even higher numbers have been suggested by meta-analyses with predicted numbers ranging from 48 to 60 million [13] to 62–72 million [14] infected individuals corresponding to 13.02% (95% CI, 11.96–14.11) and 14.57% (95% CI 12.93 to 16.27), respectively. The up to six-fold discrepancies are likely due to differences in the inclusion criteria as critically discussed by Razavi-Shearer and co-workers [15]. It should be noted that the overall prevalence reported by Stockdale et al. is highly heterogeneous. In highly endemic areas, including Mongolia, the Middle East, and parts of South America, HDV markers such as anti-HDV antibodies or circulating HDV RNA can be detected in up to 60% of HBsAg carriers [15], while in Western Europe and North America, this percentage is relatively low (3%) [12]. Transmission of HDV occurs predominantly through parenteral (blood-to-blood) exposure, whereas sexual or vertical (mother-to-child) transmission appears to play only a minor role [16]. Clinically, HDV infection occurs either as coinfection or superinfection with HBV. Coinfection of patients with HBV and HDV at the same time is most often characterized by acute hepatitis and a spontaneous viral clearance in over 90% of cases. Only a minority of coinfected individuals subsequently develop chronic HDV infection [17]. In contrast, HDV superinfection describes an HDV infection in patients with an already established chronic HBV infection. This typically leads to severe acute hepatitis and a progression to chronic hepatitis delta (CHD) occurring in up to 80% of cases (reviewed in [18]). Adaptive immune responses against HDAg are detectable, but they are often dysfunctional or escape-prone in chronic HDV infection and thus contribute to the development of chronic liver inflammation and disease progression. These aspects are only briefly discussed here but are reviewed in detail elsewhere [19,20]. This review focuses on the differences between HBV and HDV sensing by the innate immune system and the molecular mechanisms underlying HDV pathogenicity. The reviewed literature is put into perspective with recent clinical studies addressing the role of HDV in the progression of liver disease and the development of hepatocellular carcinoma (HCC).

## 2. Virus Sensing and Immune Responses

Genome replication is an essential step in the viral life cycle. To initiate this process, viruses import their genomes into host cells, exposing their foreign nucleic acid to the host’s antiviral defenses, which then mount an appropriate response (reviewed in [21]). This detection relies on pattern recognition receptors (PRRs), which sense pathogen-associated molecular patterns (PAMPs). Key PRRs include Toll-like receptors (TLRs), the cytosolic DNA sensor cyclic GMP–AMP synthase (cGAS), and retinoic acid-inducible gene I (RIG-I)-like receptors (RLRs) [22]. Their activation triggers the recruitment of downstream signaling molecules involved in innate immune signaling, such as stimulator of interferon genes (STING), TIR-domain-containing adapter-inducing interferon-β (TRIF), interferon-inducible protein 16 (IFI16), mitochondrial antiviral-signaling protein (MAVS), and myeloid differentiation primary response 88 (MyD88) [23].

Hepatotropic viruses are predominantly detected by at least one of the three RLR family members: RIG-I, melanoma differentiation-associated protein 5 (MDA5), and laboratory of genetics and physiology 2 (LGP2). These three proteins possess a C-terminal domain (CTD) that mediates RNA binding and a DExD/H-box helicase domain. RIG-I and MDA5 also contain two N-terminal caspase activation and recruitment domains (CARD1 and CARD2), which are required for MAVS recruitment, whereas LGP2 lacks these CARD domains and therefore does not directly participate in signal transduction (reviewed in [24]). RIG-I and MDA5 recognize distinct RNA species: RIG-I detects short double-stranded RNAs bearing a 5′ triphosphate [25] or diphosphate [26], while MDA5 recognizes longer double-stranded RNAs. This specificity also reflects structural differences, as the CTD of RIG-I contains a triphosphate-binding pocket and an RNA duplex end-capping loop. MDA5 lacks these features, so its recognition ability relies solely on weak interactions with internal regions of long double-stranded RNA [27].

### 2.1. HBV Sensing

Although HBV possesses a partially double-stranded circular DNA genome, it can be detected by RLRs during later stages of infection. RIG-I has been shown to recognize the epsilon stem-loop structure of pre-genomic RNA (pgRNA) from genotypes A, B, and C in primary human hepatocytes (PHH) [28]. In contrast, another study reported that while MDA5 senses HBV genotype D in transfected Huh7 cells and in mice hydrodynamically injected with an HBV replicon plasmid, RIG-I did not bind HBV-derived nucleic acids in this context [29]. These opposite findings remain puzzling, given that HBV genotypes mainly differ by substitution mutations located in the polymerase sequence or in the major hydrophilic region of HBsAg [30]. As these mutations do not alter the length of HBV pgRNA or the nature of its 5′ end, the involvement of RIG-I versus MDA5 in sensing different genotypes remains unexpected and may be linked to the different models used.

The human TLR family comprises ten members, among which TLR2 has been identified as capable of detecting components of the HBV ribonucleoprotein particle. The HBV capsid, or core protein (HBc), was shown to activate TLR2 in HEK293 cells expressing this receptor [31]. More recently, studies in PHH demonstrated that TLR2 senses HBV particles and triggers innate immune activation [32]. While the viral component responsible for this activation was not identified, HBsAg was previously shown to induce TLR2 signaling in monocytes [33]. Thus, TLR2 may recognize both HBc and HBsAg during HBV infection. Additionally, TLR4 and its co-receptor CD14 contribute to monocyte and Kupffer cell activation through interactions with HBsAg [34].

During its replication cycle, HBV produces two forms of double-stranded DNA (rcDNA, relaxed-circular DNA; cccDNA, covalently closed circular DNA), which can theoretically be detected by DNA sensors. The cGAS protein is a well-studied sensor of double-stranded DNA; cGAS/STING signaling can be activated in hepatocytes when large amounts of rcDNA are transfected. However, in an infectious context, such activation is not observed [35], likely due to the low expression of cGAS/STING in PHH. Indeed, Thomsen et al. [36] demonstrated that human and murine hepatocytes do not express detectable levels of STING. However, overexpression of STING restores the ability of hepatocytes to mount a type I interferon response upon exposure to the HBV genome. A notable exception among HBV sensing mechanisms is the detection of cccDNA by IFI16, a member of the absent in melanoma 2 (AIM2)-like receptor family. Unlike most PRRs, IFI16 is a nuclear sensor capable of recognizing double-stranded viral DNA, including cccDNA [37]. Despite being detected by multiple PRRs, HBV is considered a stealth virus, as it does not induce type I or III production of interferon nor trigger the upregulation of interferon-stimulated genes (ISGs) [38]. The absence of an efficient antiviral response underscores the virus’s capacity to block signaling cascades required for effective immunity. These evasion strategies, as well as the contributions of viral proteins, have been reviewed in detail by Chen et al. [39].

### 2.2. HDV Sensing

Unlike HBV, HDV elicits a strong innate immune response. This induction is characterized by the production of IFN-β and IFN-λ, as initially observed in differentiated HepaRG cells [40], in humanized mice coinfected with HBV/HDV [41], and in NTCP-expressing mice infected with HDV alone [42]. Shortly afterwards, Suárez-Amarán et al. [43] identified MAVS as a central mediator of IFN-β induction using an AAV-based mouse model enabling replication of both HBV and HDV genomes. Given that MAVS is a downstream effector of RLRs, the main hypothesis at the time was that the HDV genome is detected by one of these sensors. In 2018, Zhang and coworkers [44] demonstrated that MDA5 is the PRR that recognizes the HDV genome. They demonstrated that while silencing TLR3, RIG-I, or MDA5 did not affect viral replication, only MDA5 knockdown abolished interferon production in an infectious context. Furthermore, the overexpression of MDA5 was found to enhance the interferon response, particularly the expression of two ISGs known to be induced during HDV infection: radical S-adenosyl methionine domain-containing protein 2 (RSAD2) and interferon-induced protein 44 (IFI44). In 2023, the same group also established the role of LGP2 in HDV sensing, which is the most recently identified RLR member [45]. LGP2 is required to initiate the conformational changes in MDA5 necessary for RNA recognition and knock-out of LGP2 in NTCP-HepaRG cells or PHH, led to a complete loss of IFN-β, IFN-λ, and RSAD2 expression [46]. The authors also demonstrated that both the ATPase and RNA-binding domains of LGP2 are essential for interferon induction in NTCP-expressing HepaRG cells [47].

**Figure 1:**
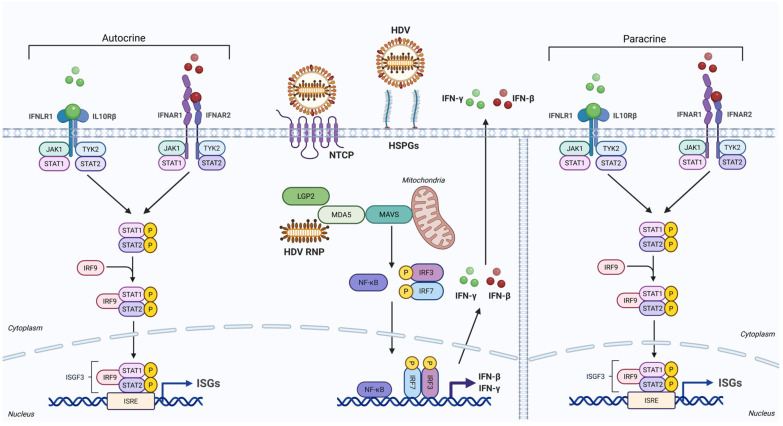
HDV sensing and interferon response. HDV enters hepatocytes by binding to heparan sulfate proteoglycans (HSPGs), followed by entry via the sodium/taurocholate co-transporting polypeptide (NTCP). After internalization, the ribonucleoprotein (RNP) is recognized by melanoma differentiation-associated protein 5 (MDA5) and laboratory of genetics and physiology 2 (LGP2). The caspase activation and recruitment domains (CARD1 and CARD2) of MDA5 mediate the recruitment of the mitochondrial antiviral signaling protein (MAVS) to initiate signal transduction that activates IFN regulatory factor 3/7 (IRF3/7) and nuclear factor-κB (NF-κB). These transcription factors translocate to the nucleus and induce the transcription of IFN-β and IFN-λ. Secreted IFNs then bind to their receptors in an autocrine and paracrine manner to activate JAK/STAT signaling. This pathway leads to the nuclear translocation of the interferon-stimulated gene factor 3 (ISGF3), which binds to interferon-stimulated response elements (ISREs) to initiate interferon-stimulated genes (ISGs) transcription. Figure created in BioRender. Lupberger, J. (2026) https://BioRender.com/7izhn5f.

The interferons produced during HDV infection act in both an autocrine and paracrine manner by binding to their cognate receptors IFNAR1/IFNAR2 for IFN-β and IFNLR1/IL10R2 for IFN-λ [48] (Figure 1). This binding activates a signaling cascade involving Janus kinases (JAK1/2), tyrosine kinase 2 (TYK2), signal transducer and activator of transcription 1/2 (STAT1/2), and IFN regulatory factor 9 (IRF9). Upon phosphorylation, STAT1 and STAT2 heterodimerize and associate with IRF9 to constitute the transcription factor ISGF3 [49]. Nuclear translocation of ISGF3 enables its binding to interferon-stimulated response elements (ISREs), thereby promoting the transcription of interferon-stimulated genes.

Despite the strong induction of ISGs, HDV is insensitive to this response. HDV is known for its ability to hijack cellular RNA polymerases to complete its replication cycle. This strategy counteracts the fact that many ISGs target viral RNA-dependent polymerases. The nuclear localization of HDV replication also impairs immune detection, as many PRRs and ISGs are mainly cytoplasmic. Even when genomic RNAs transit through the cytoplasm for virion assembly, their association with S-HDAg, L-HDAg and HBV envelope proteins avoids their recognition by cytosolic PRRs [50]. The HDV genome structure also contributes to immune evasion. Its circular genomic RNA has no free 5′ or 3′ ends and encodes only two isoforms of a single protein. Compared to other viruses, this simplicity reduces the number of viral components that PRRs or ISGs can detect or target. In addition, HDV attenuates IFN-α signaling by blocking Tyk2 phosphorylation and preventing STAT1 and STAT2 activation. This inhibition results in reduced expression of key ISGs such as Mx1, 2′,5′-OAS, and PKR [51]. Despite the insensitivity of HDV to ISGs, exogenous IFN-α impacts the HDV life cycle by activating immune cells, inhibiting early steps of infection [52] and abrogating cell-mediated propagation [53]. Surprisingly, HDV takes advantage of JAK1 by an interferon-independent mechanism to enhance ERK1/2 phosphorylation, leading to S-HDAg phosphorylation. This hijacking increases S-HDAg affinity for RNA polymerase II, thereby promoting HDV RNA transcription [54].

Compared to other chronic viral hepatitis, both HDV and hepatitis C virus (HCV) induce a strong type I/III interferon response and associated ISG expression [55,56]. However, for HCV this response is partially blunted mainly by the viral NS3/4A protease cleaving MAVS, thus dampening the associated signaling [56]. In contrast, HBV is only weakly detected by PRRs in vivo and is considered a stealth virus with largely absent ISG responses [57]. Taken together, the interferon response triggered during HDV infection is an interplay between cellular restriction factors and viral evasion strategies. Consequently, the innate immune response must be interpreted with caution because results often vary depending on the experimental model. Nevertheless, it is essential to understand these complex mechanisms, as interferon is still considered a therapeutic strategy in combination with the entry inhibitor bulevirtide [58].

In addition to innate immune evasion, HDV can also escape recognition by the adaptive immune system. A screening of CD8 epitopes in CHD patients identified only two HLA-B*27-restricted epitopes in individuals with resolved infections, which is relatively low compared to other hepatotropic viruses [59]. Furthermore, escape mutations were detected within these epitopes, enabling the virus to evade detection by CD8 T-cells. The same group identified polymorphisms in the HDV proteome associated with HLA class I alleles, further strengthening their conclusions [60]. Similarly, Kefalakes and colleagues reported the presence of escape variants leading to an altered detection by memory-like HDV-specific CD8+ T cells [61]. T cell exhaustion is important for HDV persistence, but a specific analysis of exhaustion phenotypes is hindered by the fact that the majority of detectable CD8+ T cells target epitopes are subjected to viral escape.

## 3. Molecular Basis of HDV Pathogenesis

**Figure 2:**
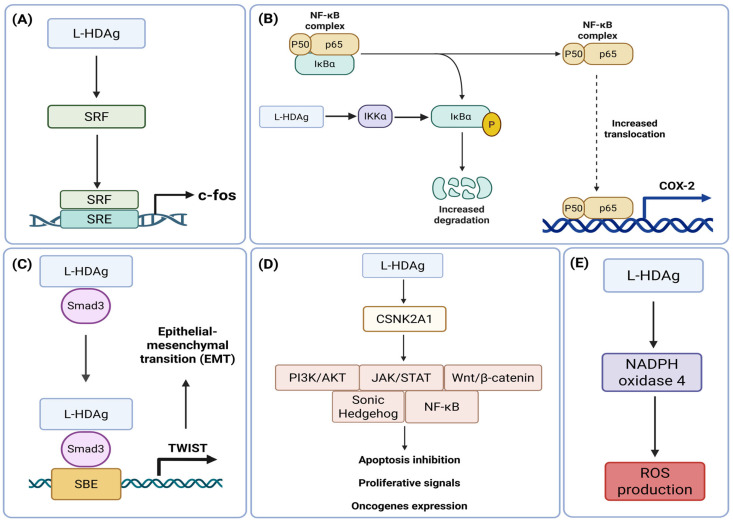
L-HDAg-associated pathogenic mechanisms. (**A**) L-HDAg increases serum response factor (SRF) expression that leads to improved recognition of serum response element (SRE) and therefore, transcription of c-fos proto-oncogene. (**B**) Upregulation of IKK-α by L-HDAg leads to nuclear factor-κB light polypeptide gene enhancer in B-cells inhibitor alpha (IκBα) phosphorylation and nuclear factor-κB (NF-κB) pathway activation. (**C**) Binding of L-HDAg to sterile alpha motif domain containing 3 (SMAD3) to activate the transcription of TWIST protein, promoting epithelial–mesenchymal transition (EMT) and consequently fibrosis. (**D**) Upregulation of casein kinase 2 alpha 1 (CSNK2A1), the catalytic subunit of casein kinase 2 (CK2). This activation participates in the dysregulation of major signaling pathways. (**E**) L-HDAg induces NADPH oxidase 4 expression, leading to increased reactive oxygen species (ROS) production. Figure created in BioRender. Lupberger, J. (2026) https://BioRender.com/ixoylxf (accessed on 1 January 2026).

CHD is widely recognized as the most severe form of viral hepatitis. Disease progression is associated with a marked increase in the risk of cirrhosis, liver decompensation, and mortality compared with HBV monoinfection [62]. However, the direct and indirect mechanisms contributing to HDV-induced severe liver disease and cancer remain elusive. Numerous studies have been carried out to determine how HDV induces fibrosis, cirrhosis and potentially HCC, as well as which viral components are involved. The large isoform of the delta antigen has attracted early attention in the field due to its role in the dysregulation of cellular homeostasis. In a study published in 2000, Goto et al. [63] demonstrated that L-HDAg activates serum response factor (SRF)-associated transcription. The aim of this study was to elucidate the respective roles of both HDAg isoforms in five signal transduction pathways: SRF-, serum response element (SRE)-, nuclear factor-κB (NF-κB)-, activator protein 1 (AP-1)-, and cyclic AMP response element (CRE)-dependent pathways. The authors demonstrated that L-HDAg induces c-fos expression by enhancing SRF transcriptional activation, allowing SRF to recognize the SRE within the c-fos promoter (Figure 2A). However, SRF DNA-binding affinity was similar in the presence and absence of L-HDAg, and no direct interaction between L-HDAg and SRF was detected by immunoprecipitation. These results should therefore be interpreted with caution, as this early study relied on synthetic promoters and L-HDAg overexpression. To date, no further evidence supporting a role of L-HDAg in SRF-associated signaling pathways has been published. In addition, the authors did not observe any effect of L-HDAg on NF-κB signaling, although several later studies established a connection between L-HDAg and NF-κB-associated pathways. For instance, Huang et al. showed that L-HDAg nuclear translocation correlates with endoplasmic reticulum stress and NF-κB activation [64]. Also, Park and colleagues published a study demonstrating that L-HDAg enhances TNF-α-induced NF-κB activation in hepatic cells [65]. They observed an increased nuclear translocation of the p65 subunit due to NF-κB light polypeptide gene enhancer in B-cells inhibitor alpha (IκBα) degradation, leading to the upregulation of NF-κB target genes such as cyclooxygenase 2 (*COX-2*). This activation presumably represents a key element of HDV pathogenesis and potentially tumorigenesis, as persistent NF-κB signaling is strongly associated with HCC progression [66]. Moreover, *COX*-*2* transgene expression promotes HCC initiation by inducing promoter hypermethylation through Tet methylcytosine dioxygenase 1 (TET1) reduction, silencing tumor-suppressive genes such as latent transforming growth factor beta binding protein 1 (*LTBP1*) and activating major oncogenic pathways including mechanistic target of rapamycin (mTOR) and AKT signaling [67]. In addition, a recent study observed a strong upregulation of IKK-α in L-HDAg-expressing cells, which results from IκBα phosphorylation and NF-κB pathway activation [68]. This finding corroborates the strong impact of L-HDAg on NF-κB-associated pathways (Figure 2B).

Beyond NF-κB signaling, L-HDAg also influences transforming growth factor-β (TGF-β) signaling. Choi and colleagues demonstrated in 2007 that L-HDAg strongly activates TGF-β and AP-1 signaling pathways and increases the protein expression of plasminogen activator inhibitor-1 (PAI-1) [69]. These findings strongly support a role of L-HDAg in liver fibrosis and cirrhosis, as PAI-1 inhibits urokinase plasminogen activator (uPA). uPA is responsible for converting plasminogen into plasmin and thereby mediating extracellular matrix (ECM) degradation [70]. These findings were further consolidated in 2020 by Liang and coworkers [71] adding additional details to the understanding of L-HDAg-mediated TGF-β signaling. Their study revealed that L-HDAg binds to sterile alpha motif domain containing 3 (SMAD3) and activates the expression of twist basic helix-loop-helix transcription factor (TWIST) through a Smad3-binding element (SBE) located in the proximal TWIST promoter region. Mutation of the SBE or knockdown of *SMAD3* reduced L-HDAg-mediated TWIST promoter activation, indicating a specific regulatory mechanism (Figure 2C). This signaling cascade results in increased TGF-β secretion, upregulation of AKT serine/threonine kinase 2 (AKT2), downregulation of neurofibromin 1 (NF1), and enhanced expression of fibrosis-promoting TWIST downstream target genes, including serpin family E member 1 (*SERPINE1*) coding for PAI-1, as previously shown by Choi et al. [69], and TIMP metallopeptidase inhibitor 1 (*TIMP1*). Dysregulation of these genes drives epithelial–mesenchymal transition (EMT), leading to the generation of fibroblast-like cells that accumulate and promote progressive fibrosis [72]. This study also emphasizes the carcinogenic potential of HDV, as the same group previously demonstrated that TWIST expression and EMT promote HCC metastasis [73].

More recently, a kinome profile in Huh7 cells expressing L-HDAg, S-HDAg, or the HDV genome was published, providing a global overview of dysregulated signaling pathways. The authors of this study identified more than 90 kinases significantly altered by viral components, especially L-HDAg, likely due to its higher phosphorylation level [68]. Indeed, L-HDAg is phosphorylated about ten times more than S-HDAg [74]. Interestingly, casein kinase 2 alpha 1 (CSNK2A1), the catalytic subunit of casein kinase 2 (CK2), was among the most upregulated kinases. CK2 is implicated in the dysregulation of major signaling pathways such as PI3K/Akt, NF-κB, JAK/STAT, Wnt/β-catenin, and Hedgehog signaling (Figure 2D) [75]. CK2 has also been suggested to promote increased proliferative capacity and tumor growth in cholangiocarcinoma cells [76]. Furthermore, Williams et al. [77] and Smirnova et al. [78] demonstrated that L-HDAg expression induces NADPH oxidase 4 expression, leading to increased reactive oxygen species (ROS) production (Figure 2E). Elevated ROS levels promote liver disease progression to cancer [79] and are known to activate the PI3K/Akt pathway [80], which further highlights the heterogeneity of cellular processes triggered by L-HDAg.

**Figure 3:**
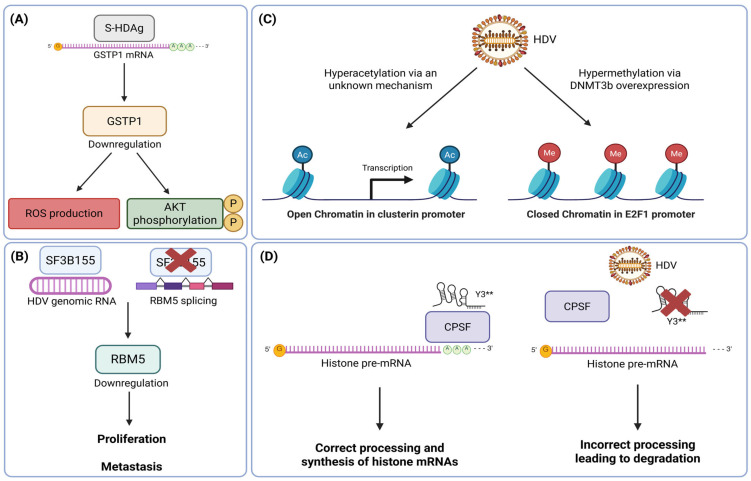
Pathogenic mechanisms independent of L-HDAg. (**A**) S-HDAg can bind to the glutathione S-transferase Pi 1 (*GSTP1*) mRNA and impair its translation, leading to a decrease in GSTP1 protein level. This downregulation is involved in ROS production and AKT activation. (**B**) HDV genomic RNA can interact with the splicing factor SF3B155 and perturb SF3B155-dependent gene splicing, such as the tumor suppressor RNA binding motif protein 5 (RBM5). (**C**) HDV induces histone hyperacetylation near the clusterin promoter, leading to clusterin overexpression and hypermethylation by DNA (cytosine-5)-methyltransferase 3 beta (DNMT3b) overexpression, which results in a downregulation of E2F1. (**D**) The long-non-coding RNA (lncRNA) Y3 is suppressed in HDV-associated hepatocellular carcinoma (HCC). Y3**, a truncated form of Y3, interacts with the cleavage and polyadenylation specificity factor (CPSF) to regulate histone pre-mRNA processing. Depletion of Y3 potentially impairs histone pre-mRNA and mRNA synthesis. Figure created in BioRender. Lupberger, J. (2026) https://BioRender.com/xrino7q (accessed on 1 January 2026).

But L-HDAg is not the only direct contributor to HDV pathogenicity. Also, S-HDAg potentially contributes to liver damage independently of L-HDAg [81] and thus deserves more attention. However, so far, the pathogenic mechanisms mediated by S-HDAg are still poorly characterized, as this isoform has mainly been studied for its role in viral transcription and replication. S-HDAg has been shown to induce liver injury by binding to glutathione S-transferase Pi 1 (*GSTP1*) mRNA and significantly downregulating GSTP1 protein expression [82]. Consequently, GSTP1 downregulation leads to ROS accumulation, as this enzyme plays a critical role in detoxification and the reduction in oxidative stress [83]. GSTP1 also functions as a tumor suppressor by inhibiting cell proliferation through reduction in Akt phosphorylation [84]. Furthermore, GSTP1 downregulation via promoter hypermethylation is correlated with HCC incidence and poor clinical outcomes [85]. Collectively, these findings suggest that S-HDAg may also contribute to HDV-associated carcinogenesis by reinforcing GSTP1 repression (Figure 3A).

More generally, HDV infection leads to the downregulation of other tumor suppressor genes, such as p53 in transfected HEK-293 cells [86], although the mechanism by which HDV affects p53 expression remains unknown. In 2020, Tavanez et al. [87] revealed an interaction between the splicing factor SF3B155 and HDV genomic RNA. SF3B155 is a component of the U2 snRNP complex and is essential for the early recognition of 3′ splice sites in human pre-mRNAs. This interaction leads to the hijacking of SF3B155 and induces alterations in the splicing of human genes whose alternative splicing is SF3B155-dependent, such as the tumor suppressor RNA binding motif protein 5 (RBM5) (Figure 3B). HDV expression results in a significant reduction in RBM5 protein levels, which may contribute to early progression toward HCC, as RBM5 overexpression can suppress the proliferation and metastatic formation of colorectal cancer cells [88]; however, a tumor-suppressing role of RBM5 in HCC remains to be confirmed.

HDV modulates gene expression as well through epigenetic mechanisms. Expression of S-HDAg and L-HDAg has been associated with elevated histone H3 acetylation, notably at the clusterin promoter in Huh7 cells (Figure 3C) [89]. Moreover, clusterin overexpression enhances cell survival and has been reported in up to 89% of human HCC cases [90]. This increase is also linked with metastatic HCC when compared with primary tumors [91]. Therefore, the ability to regulate the expression of clusterin by HDV may contribute to the long-term development of liver damage and may represent only the tip of the iceberg in epigenetic dysregulation of the host genome. On the DNA methylation level, HDV influences the expression of DNA methyltransferases, particularly DNA (cytosine-5)-methyltransferase 3 beta (DNMT3b), which is responsible for *de novo* DNA methylation through STAT3 activation [92]. DNMT3b overexpression leads to hypermethylation of the E2F1 promoter (Figure 3C). The E2F1 transcription factor binds preferentially to the retinoblastoma protein (pRB) in a cell cycle–dependent manner and thus can mediate both cell proliferation and p53-dependent or -independent apoptosis [93]. E2F1 thereby plays a critical role in cell cycle control and tumor suppression. These findings suggest that HDV may contribute to HCC development, at least in part, through alterations in DNA methylation. This may also indicate a role for HDV in cell cycle regulation. The same study also performed cell cycle analyses comparing mock-transfected and HDV-transfected cells and demonstrated that HDV induces G2/M cell cycle arrest [92]. Subsequent studies confirmed its role in cell cycle dysregulation. For example, a 2021 study compared cancerous and paracancerous specimens from patients with HBV- or HDV-associated HCC with the aim of identifying genes closely related to HDV-associated HCC [94]. The authors identified seven potential candidate genes (CDC6, CDC45, CDCA5, CDCA8, CENPH, MCM4, and MCM7) that are significantly associated with dysregulation of cell cycle and DNA replication. Moreover, they are involved in multiple pathways related to HCC development and poor prognosis [95,96,97]. In the same notion, the mentioned kinome profiling by Thiyagarajah et al. [68] revealed that HDV replication and expression of both HDAg lead to inhibition of CDK4/6, inducing G2/M phase cell cycle arrest. Constitutive CDK4/6 inhibition could therefore promote the accumulation of mutations that enable escape from cell cycle arrest and apoptosis, ultimately contributing to HCC progression.

Another epigenetic layer involves long-non-coding RNAs (lncRNAs), which have been shown to play important roles in liver pathogenesis and cancer [98,99]. Interestingly, the lncRNA Y3 is specifically suppressed in HDV-associated HCCs [100] compared to other etiologies. This finding is particularly intriguing because Y3 overexpression has been reported in bladder, cervical, colon, kidney, lung, and prostate cancers, where it regulates cell proliferation [101]. Köhn et al. [102] demonstrated that the truncated form of Y3 called Y3** binds to the cleavage and polyadenylation specificity factor (CPSF) and that Y3** associates with the 3′ untranslated region (UTR) of histone pre-mRNAs. They showed that depletion of Y3** impairs 3′ end processing of histone pre-mRNAs as well as the formation and protein dynamics of histone locus bodies, where histone mRNA synthesis and processing occur (Figure 3D). It is therefore tempting to speculate that an HDV-induced downregulation of Y3 could lead to abnormal histone expression and thereby may contribute to HDV-associated carcinogenesis.

## 4. Pathogenesis of Chronic HDV Infection in Patients

Numerous case–control and cohort studies have been conducted since the discovery of HDV, which are essential clinical data sources to understand HDV pathogenesis. However, depending on the study design and inclusion criteria, some of the conclusions drawn may differ. Besides direct pathogenic molecular mechanisms in infected hepatocytes, HDV infection leads to drastic changes in the immune cell landscape of patients with CHD. Mucosa-associated invariant T (MAIT) cells and natural killer (NK) cells appear to be among the most impacted population during chronic HDV infection. For MAIT cells, Dias and colleagues reported a significant reduction of this population in both peripheral blood and liver tissue of HDV-infected patients. This was accompanied by an increased secretion of the pro-inflammatory cytokines IL-12 and IL-18 promoting MAIT cells apoptosis [103]. A later study confirmed that the number of MAIT and NK cells in the livers of HDV-infected patients is lower than in uninfected controls [104]. Interestingly, MAIT cells and NK cells are activated and degranulated more frequently in the liver than in the blood, suggesting a strong intrahepatic immune response that causes severe liver damage [104]. It was demonstrated that during CHD infection, the inflammatory NK cell response lacks negative feedback regulation due to the downregulation of the T cell immunoreceptor with Ig and ITIM domains (TIGIT) [105]. TIGIT is essential to prevent and control NK cell–mediated liver damage during acute viral hepatitis [106]. Its reduced expression therefore promotes liver damage and contributes to the establishment of an inflammatory environment. In addition to NK and MAIT cells activation, HDV leads to the accumulation of HDV antigen-unspecific T cells. For instance, HBV/HDV coinfection induces a high expression level of CXCL9-11 chemokines, which are responsible for the recruitment of HBV/HDV-unspecific CD4 T cells expressing CXCR3 [107]. Furthermore, antigen-nonspecific activation of liver-resident CD8+ T cells has been reported in HDV infected patients [104]. Collectively, these mechanisms are non-negligible contributors to the establishment of an inflammatory state and the rapid progression of liver disease and thus, the adaptive immune response to HDV is part of the multifactorial process that increases the risk of HCC in CHD patients.

Early studies suggested that the incidence of HCC in patients with CHD was comparable to that observed in CHB [108,109]. However, more recent clinical studies have provided evidence supporting a significant contribution of HDV in carcinogenesis. In a prospective cohort of 299 patients followed for a mean duration of 233 months, Romeo et al. revealed a significant association of HDV replication with HCC occurrence in this cohort [110]. The authors confirmed their findings in an additional cohort of 193 patients. They found that high HDV RNA levels in the blood serum were indeed significantly associated with cirrhosis progression and HCC [111]. Interestingly, Brancaccio and colleagues showed that HDV infection increased the risk of HCC in patients achieving HBV DNA suppression under treatment with nucleos(t)ide analogs [112]. In their study, the rate of death or liver transplantation was 2.92 per 1000 patient-months in HDV-infected individuals versus 0.38 in HBV monoinfected patients (*p* < 0.001), liver decompensation occurred at rates of 1.53 versus 0.13 (*p* = 0.015), and HCC incidence was nearly threefold higher in the HDV cohort (3.12 vs. 1.12; *p* = 0.02).

To shed more light on the existing clinical data, Alfaiate and co-workers performed a meta-analysis of the current literature in 2020 [113], comparing 93 studies conducted across 36 countries (68 case–control studies involving 22,862 patients and 25 cohort studies including 75,427 patients). These results showed a significantly increased risk of HCC in patients with CHD compared with those with HBV monoinfection. However, a genotype-specific analysis was precluded by insufficient data. This meta-analysis provided an important indicator that HDV itself contributes to elevated liver cancer risk in patients, based on available data until 2019. To complement and update this finding from recent studies, the following section will provide an overview of post-2020 studies.

Since 2020, several studies have confirmed the implication of HDV RNA accumulation in liver disease severity, but again, the impact of HDV on HCC risk remains blurry. Furquim d’Almeida et al. [114] demonstrated that a positive HDV RNA status in patients coinfected with HBV and HDV is associated with a higher likelihood of developing HCC (*p =* 0.003). Similar associations were observed for liver decompensation (*p* = 0.002) and liver transplantation (*p* = 0.027). Nonetheless, a long-term study performed in Sweden on the impact of viremia underlined the importance of HDV RNA viremia in liver-related events. The authors compared 337 patients with anti-HDV positivity at baseline with a mean follow-up of 6.5 years (from 0.5 to 33.1 years). This cohort included 233 patients with HDV RNA viremia and 91 without HDV viremia. Their data indicated a 3.8-fold higher risk for liver-related events and a trend of 2.6-fold higher risk for HCC in patients with HDV viremia, compared to patients without HDV viremia [115]. It should be noted, however, that the results for HCC were not statistically significant (*p* = 0.23). Also, the authors could not identify any significant independent factors that are associated with the development of cirrhosis or liver-related events among patients without cirrhosis. This lack of statistical significance may be explained by the low incidence of such events. A Canadian retrospective study compared 135 HDV seropositive patients with 5132 patients with an HBV monoinfection [116]. They observed that complications of end-stage liver disease, such as liver transplantation (6.7% vs. 0.1%; *p* < 0.001), variceal bleeding, and hepatic encephalopathy, were significantly more frequent in the HDV cohort than in the HBV monoinfected cohort. In addition, the study revealed an important increase in cirrhosis (45.2% vs. 3.2%; *p* < 0.001) and HCC (8.2% vs. 1.0%; *p* < 0.001) rates between the cohorts. The authors also dissected the role of HDV RNA level by stratifying the HDV cohort into 2 groups: HDV RNA-positive (*n* = 95) and HDV RNA-negative (*n* = 35). HDV RNA positivity was associated with cirrhosis development (53.7% vs. 17.1%; *p* < 0.001), elevated alanine aminotransferase, aspartate aminotransferase, transient elastography, but not with HCC formation (8.4% vs. 5.7%; *p* > 0.999). These data confirm previous results demonstrating the implication of HDV RNA in liver disease except for HCC formation. However, the comparison between HDV patients and HBV monoinfected patients showed a significant increase in HCC development. The authors also conducted a genotype analysis in this cohort. However, no association was revealed between HDV genotypes and the clinical outcomes. Yet, this finding must be interpreted with caution, as the analysis is limited by the small sample size.

Early phylogenetic studies grouped HDV into 8 genotypes, which can replicate with 10 different HBV genotypes in patients [117,118]. As HDV replication depends on host RNA polymerases with lack of fidelity, HDV is considered as quasispecies with evidence for infections with mixed HDV genotypes [119]. Indeed, HDV genotype–specific pathogenicity remains poorly understood, also because most sequence and clinical studies are largely dominated by genotype 1 [10,120], despite evidence suggesting a poorer prognosis associated with certain genotypes [121]. For instance, a nationwide retrospective study performed by the French National Reference Centre for HDV on 1112 HDV-infected patients showed a heterogeneous distribution of HDV genotypes with differences in clinical outcomes [122]. The genotypes found in the cohort were HDV-1 (75.9%), HDV-5 (17.6%), HDV-7 (2.9%), HDV-6 (1.8%), HDV-8 (1.6%), HDV-2 (0.2%) and HDV-3 (0.1%). HDV-1 and HDV-5 were associated with a higher risk of developing cirrhosis. Interestingly, HCC occurrence was also associated with detectable HDV RNA (hazard ratio, 2.14; *p* = 0.01), further adding to the evident association of HDV replication with HCC risk. HDV-2, however, is associated with a milder liver disease with sub shadings within local variants in Japan [123]. A comprehensive interpretation of HDV genotype-specific clinical impact is further complicated by contributions from HBV genotypes and sex-based differences. While HDV-infected females tend to develop cirrhosis earlier, males generally exhibit more advanced liver disease alongside multiple comorbidities [124]. During mono-infection, HBV genotype C, D and F patients have a higher risk of cirrhosis and HCC than other HBV genotypes, leading to a poorer clinical outcome [125,126]. However, as HDV co-infection suppresses HBV replication (reviewed in [127]), the clinical impact of HBV genotypes during HDV co-infection in patients may be attenuated.

So, is the HCC risk elevated mainly due to the accelerated cirrhosis in patients? To decipher whether HDV has a direct or indirect role in HCC development, a study compared 175 patients with CHD-related cirrhosis to 175 patients with CHB-related cirrhosis. Interestingly, they showed that CHB patients developed HCC more frequently than CHD patients (35.5% vs. 18.5%) [128]. This finding was unexpected, as cirrhosis is a dominant intrinsic risk factor for HCC development. If HDV is not involved in HCC development once cirrhosis is established, HCC rates would be expected to be similar between CHB and CHD groups rather than twofold higher in CHB patients. One explanation could be interferon (IFN) treatment: only 11.4% of CHB patients were treated with IFN-α compared to 38.3% of CHD patients. The HCC-protective role of IFN is still debated; in this study, they observed more benign clinical long-term outcomes with IFN-based therapy. These findings may support an indirect role of HDV in HCC risk based on rapid progression to cirrhosis. However, evidence supporting a direct carcinogenic role of HDV was provided recently by Roulot et al. in a cohort of 142 HBV–HDV coinfected and 271 HBV monoinfected patients with cirrhosis [129]. In this study, HDV coinfection was identified as an independent factor associated with HCC incidence (hazard ratio, 11.99; 95% CI, 2.53–56.76; *p* = 0.002) after multivariate adjustment for age, overweight, tobacco consumption, and other factors.

Taken together, the data presented in this review, along with evidence reported elsewhere, have led to the classification of HDV as a Group 1 carcinogenic agent by the International Agency for Research on Cancer (IARC) in 2025 [130]. Therefore, HDV has joined HBV and HCV, which have been present in this group since 2009.

## 5. Concluding Remarks

CHD represents the most severe form of chronic viral hepatitis and is consistently associated with rapid progression toward advanced liver disease, including cirrhosis, liver decompensation, and increased mortality compared with HBV monoinfection. Despite nearly five decades of research, pathogenic mechanisms underlying HDV–associated liver injury and its contribution to HCC development still need investigation. This review summarizes key molecular and clinical data to understand HDV pathogenesis and its role in liver carcinogenesis. An important characteristic of HDV infection is its strong immunogenicity. Unlike HBV, which evades innate immune detection, HDV is efficiently sensed by MDA5 and LGP2, leading to the induction of type I and III interferons and production of numerous ISGs. Nevertheless, this antiviral response fails to inhibit HDV replication and propagation. This is due to the remarkable capacity of HDV to evade innate immune restriction through nuclear replication, protection of viral RNA by HDAgs, reliance on host RNA polymerases, and active inhibition of interferon signaling. The resulting state constitutes a major cause of chronic inflammation. The strong and untampered innate immune response combined with altered adaptive immune responses induced by HDV are main contributors to faster fibrogenesis, higher levels of chronic necroinflammation, and a more pronounced risk of HCC development. However, other more indirect contributors are evident as we also learned from the stealth virus HBV, where important profibrotic events are induced independently of the immune response [131,132,133].

As summarized in this review, experimental evidence indicates that HDV directly perturbs cellular homeostasis through specific pathways. L-HDAg has appeared as a key factor capable of dysregulating signaling cascades implicated in fibrosis and tumorigenesis. Activation of NF-κB signaling, oxidative stress, and stimulation of TGF-β–dependent EMT converge to promote extracellular matrix accumulation and liver fibrosis. L-HDAg induces important alterations in kinase activity, including upregulation of CK2 and IKKα, affecting major oncogenic pathways such as PI3K/Akt, NF-κB, and JAK/STAT signaling. Also, S-HDAg contributes to liver injury and carcinogenesis. By downregulating GSTP1 expression and promoting ROS accumulation, S-HDAg amplifies oxidative stress and impairs tumor suppressor functions. In parallel, HDV induces epigenetic and post-transcriptional alterations, including changes in histone acetylation, DNA methylation, RNA splicing, and non-coding RNA expression, that affect key regulators of cell cycle control, apoptosis, and genomic stability. A limitation of many mechanistic studies, however, is the limited comparability of the used experimental models, which are often based on the overexpression of delta antigens and the absence of HBV.

Also, clinical studies investigating the association between CHD and HCC must be carefully interpreted as conclusions differ depending on the study design. Early cohorts suggested comparable HCC incidence between CHD and CHB patients, questioning the independent carcinogenic role of HDV. However, more recent prospective studies, national cohorts, and meta-analyses have clearly demonstrated elevated HDV-associated HCC risk in patients. A recurrent observation is the strong association between detectable HDV RNA and disease severity, including fibrosis, liver decompensation, and mortality. Nevertheless, whether HDV replication independently drives HCC development or primarily acts by accelerating progression to cirrhosis has remained difficult to ascertain. Several studies suggest that HDV RNA viremia is a major determinant of liver-related events, while its direct contribution to HCC risk is more difficult to establish due to limited event numbers and heterogeneity in study design. In some cohorts, HDV infection was strongly associated with cirrhosis but not with HCC occurrence, whereas in others, it remained significantly associated with increased incidence of HCC even after adjustment for classical risk factors. Notably, a recent study with only cirrhotic patients has identified HDV coinfection as an independent predictor of HCC. However, due to wide confidence intervals, these findings need cautious interpretation and require confirmation in larger, well-controlled studies. One significant gray area remains the role of the different genotypes in the progression of liver disease. Some studies have attempted to highlight disparities in pathogenicity among the eight genotypes, but these studies are often limited by the small number of patients and the lack of diagnosis.

Mechanistic and epidemiological data have led to the recent classification of HDV as a group 1 carcinogenic agent by the International Agency for Research on Cancer. HDV is now recognized as a major etiological agent in liver carcinogenesis. Experimental and clinical studies summarized in this review demonstrate direct and indirect pathogenic mechanisms of chronic inflammation, epigenetic alterations, cell cycle dysregulation, and inhibition of tumor suppressors. But what is the main cancer-inducing risk factor during CHD? So far, clinical evidence points towards the HDV-induced acceleration of liver cirrhosis. This is shared with other liver disease etiologies like chronic hepatitis C virus infection, metabolic dysfunction-associated steatohepatitis, and alcohol, where HCC arises from cirrhotic livers in most cases. However, given the coinfection with another oncogenic virus, HBV, the mostly unknown timelines of chronic HBV and the subsequent HDV superinfection in patients, the direct contributions of HDV itself are largely dominated by the oncogenic pressure of liver cirrhosis. From a clinical perspective, these findings reinforce the need for intensified HCC surveillance in patients with chronic hepatitis D, particularly those with active viral replication and liver cirrhosis. It also highlights the importance of achieving durable HDV RNA suppression with emerging antiviral therapies and limiting liver disease progression by future chemopreventive strategies complementing antiviral therapies.

## Data Availability

No new data were created or analyzed in this study. Data sharing is not applicable to this article.
